# Electrospun Hybrid Films for Fast and Convenient Delivery of Active Herb Extracts

**DOI:** 10.3390/membranes12040398

**Published:** 2022-04-01

**Authors:** Shiri Guo, Wenlai Jiang, Liangfei Shen, Gaoyi Zhang, Yiman Gao, Yaoyao Yang, Deng-Guang Yu

**Affiliations:** 1 School of Materials and Chemistry, University of Shanghai for Science and Technology, Shanghai 200093, China; 1935023610@st.usst.edu.cn (S.G.); 203613110@st.usst.edu.cn (W.J.); 1935023519@st.usst.edu.cn (L.S.); 1935040304@st.usst.edu.cn (Y.G.); 2 School of Optical-Electrical and Computer Engineering, University of Shanghai for Science and Technology, Shanghai 200093, China; 1935040829@st.usst.edu.cn; 3 Shanghai Engineering Technology Research Center for High-Performance Medical Device Materials, Shanghai 200093, China

**Keywords:** medicated film, hybrid film, herb medicine, electrospinning, drug delivery, fast dissolution

## Abstract

Herb medicines are popular for safe application due to being a source of natural herbs. However, how to deliver them in an efficacious and convenient manner poses a big challenge to researchers. In this study, a new concept is demonstrated that the electrospun polymer-based hybrid films can be a platform for promoting the delivery of a mixture of active herb extract, i.e., Lianhua Qingwen Keli (LQK), also a commercial traditional Chinese patent medicine. The LQK can be co-dissolved with the filament-forming polymeric polyvinylpyrrolidone K60 and a sweeter sucralose to prepare an electrospinnable solution. A handheld electrospinning apparatus was explored to transfer the solution into solid nanofibers, i.e., the LQK-loaded medicated films. These films were demonstrated to be composed of linear nanofibers. A puncher was utilized to transfer the mat into circular membrane a diameter of 15 mm. Two self-created methods were developed for disclosing the dissolution performances of the electrospun mats. Both the water droplet experiments and the wet paper (mimic tongue) experiments verified that the hybrid films can rapidly disintegrate when they encounter water and release the loaded LQK in an immediate manner. Based on the reasonable selections of polymeric excipients, the present protocols pave a way for delivering many types of active herb extracts in an effective and convenient manner.

## 1. Introduction

During the past half a century, polymers have acted as a backbone role to support the fast developments of pharmaceutics [1,2,3,4,5,6,7,8], particularly the dosage forms for drug delivery [9,10,11,12,13,14,15,16]. The numbers in which natural and synthetic polymers are introduced into this interdisciplinary field is always increasing [17,18,19,20]. Meanwhile, the techniques, the related methods and strategies for transferring a certain drug to a polymer-based medicated product are simultaneously expanded [21,22,23,24,25,26]. Thus, it is common sense that the joint efforts of advanced materials and advanced technologies have pushed the progress of effective, safe and convenient drug delivery.

As an important and also ancient section of medicine, Chinese herb medicine and the related Chinese patent medicine are modernized along different directions, such as effective extraction of the active ingredients from the herbs, combined applications with Western medicines and convenient and efficacious delivery of the multiple active ingredients contained in Chinese patent medicine [27,28]. Thus, it is not strange that nanomaterials and nanotechnologies have played important roles in modernizing traditional Chinese medicine [29,30]. Among different sorts of nanomaterials, polymer-based medicated films are one of the most popular ones [31,32,33,34], and show some advantages over other nanoproducts such as nanocrystals, nanolipids and inorganic nanocomposites in terms of processability, availability and feasibility [35,36,37,38,39,40].

Polymer-based medicated films can be presented in the form of round or concave particles, sheets, beads-on-a-string, dumbbells, tadpoles, rods and also linear fiber films [41,42,43,44,45,46,47]. Among these morphologies, nanofibers are a special one due to their one-dimensional format, i.e., diameter at a nano scale but with a size of length at macro scale [48,49,50,51,52,53,54]. To date, over 95% of the reported nanofibers are produced using electrospinning, which mainly present in a non-woven film [55]. As a popular electrohydrodynamic atomization (EHDA) method, electrospinning is similar to electrospraying in taking advantage of the easy interactions between working liquids and higher voltage electrostatic energy [56,57,58,59,60,61,62]. Their differences lie in the following: (1) the working fluids utilized in electrospinning often have a higher concentration and related viscosity than those exploited during electrospraying, and correspondingly (2) the products from electrospinning are fibers, whereas those from electrospraying are particles [63,64,65,66,67]. With either electrospinning or electrospraying, the functional ingredients can be co-dissolved with the polymeric matrices to experience the working processes for generating the desired polymer-based composites in a single step and a straightforward manner.

Often, a technique can be improved or developed along three directions, i.e., “practical, convenient and effective”. Electrospinning is now, on the one hand, moving forward from the single-fluid process [68,69,70,71,72] to coaxial [73,74,75,76,77,78], triaxial [79,80], side-by-side [81,82,83,84] and their combinations of multiple-fluid processes [85,86,87,88] for increasing its practicability and effectiveness of producing more kinds of complex nanostructures and the related multiple functional nanoproducts. On the other hand, electrospinning is increasing its easiness and feasibility for creating nanofibers on a large scale for possible commercial products [89,90]. Thus, the convenience of electrospinning and direct functional application of nanofibers comprises one of the important concerns in this field. Handheld electrospinning is one of the alternative solutions to this concern.

The Chinese patent medicine “Lianhua Qingwen Keli” (LQK) can be used for the treatment of influenza, particularly those patients with fever, cold aversion, muscle pain, nasal congestion and runny nose, cough, headache, dry throat and sore throat, red tongue and yellow or greasy coating. It can also be utilized for treating Coronavirus Disease 2019 (COVID-19) patients with fever, cough and fatigue [91]. However, LQK has a bitter taste, and has several adverse reactions, such as adverse gastrointestinal reactions, nausea, diarrhea, vomiting, abdominal pain, abdominal distension, dry mouth, rash, itching and dizziness [91]. Thus, new techniques for transferring LQK should be aimed to be more effective and compliant for drug delivery to the patients. Compared with traditional dosage forms, electrospun medicated nanofibers, often collected as a non-woven film, have a series of advantages for providing effective and convenient drug delivery. These advantages include small diameters of nanofibers, huge surface of the films and 3D web structures with high porosity [2,27,76]. Meanwhile, these advantageous properties can act synergistically to promote the fast dissolution of the loaded drug molecules [92,93].

In this work, a handheld electrospinning process is developed for a concept proof of the modernization of traditional Chinese medicine. With polyvinylpyrrolidone (PVP) as a filament-forming polymeric matrix and the commercial Chinese patent medicine product LQK as a model medicine composed of active herb extracts, a new type of medicated film in the form of electrospun films was generated using the handheld electrospinning process. The films were characterized using optical microscopy, scanning electron microscope (SEM) to detect their morphologies, X-ray diffraction (XRD) and Fourier-transform infrared (FTIR) to measure the components’ physical state, and two methods were developed to evaluate the functional performances of the prepared LQK-loaded hybrid films. The results demonstrated that the electrospun hybrid films can be a potential candidate for the fast and convenient oral delivery of active herb extracts.

## 2. Experimental

### 2.1. Materials

Polyvinylpyrrolidone K60 (PVP K60, Mw = 360,000, CAS No. 84057-81-8) and sucralose (CAS No. 56038-13-2) were purchased from Sigma-Aldrich Corp. (Shanghai, China). Lianhua Qingwen Keli (LQK, brown particles) was commercial products from a local pharmacy (Lao-Bai-Xing Big Pharmacy, Shanghai, China). Water (both for preparing electrospinnable fluid and for fast disintegrating experiments) was double distilled just before use.

### 2.2. Preparation of Electrospun Films

The working fluid was a blending solution of PVP K60, LQK and sucralose. The concentrations (g/g) were 10%, 5% and 1% in distilled water, respectively.

HHE-1 handheld electrospinning apparatus (Qingdao Junada Technology Co., Ltd., Qingdao, China) was exploited for the preparation of electrospun films. The apparatus is easy to operate, safe and reliable, and has high integrity. It realizes the in situ real-time spinning of electrospun nanofibers, and at the same time strengthens the design of electrostatic safety protection and ergonomics.

A cardboard wrapped with aluminum foil was used as a plate fiber collector. The distance between the nozzle of spinneret and the collector was about 15 cm. The resultant nanofiber mats were stored in a vacuum dryer (DZF6090, Shanghai Precision Instrument Co., Ltd., Shanghai, China) to reach a constant weight. The ambient temperature and relative humidity were 21 ± 3 °C and 43 ± 5%, respectively.

### 2.3. Characterizations of Nanofibers

The resultant nanofibers’ morphologies were assessed using a field-emission scanning electron microscope (FESEM, Hitachi, Tokyo, Japan). A little patch was cut from the electrospun nanofiber membranes and was adhered on the conductive tape on a sample stage. Later, the samples were coated with a thin layer of Pt through a sputter for 1.5 min under a N_2_ atmosphere. Additionally, an optical microscope (WMS-3590, Shanghai Wu-Mo Optical Instrument Corp., Shanghai, China) was utilized for the assessment of resultant nanofibers. The sampling was realized by putting a slide glass under the handheld apparatus for several minutes.

X-ray patterns of the LQK, PVP, sucralose and their electrospun hybrid nanofibers were achieved using a high-resolution X-ray diffraction system (XRD, Bruker-AXS, Karlsruhe, Germany), in which CuKα is explored as the radiation source. The experimental conditions include 45 kV, 40 mA, 1.5 Å and a θ range from 10° to 60°.

Fourier-transform infrared analyses were conducted using a spectrophotometer (FTIR, Spectrum 100, Billerica, MA, USA). All spectra were obtained at room temperature within a wavenumber range of 600–4000 cm^−1^ with acquisition of 16 scans and 2 cm^−1^ resolution.

### 2.4. Functional Performances

Two self-created methods were explored to evaluate the fast-disintegrating and fast-dissolving processes of the prepared orodispersible films. A puncher was exploited to transfer the electrospun mats into the orodispersible films. One method was to drip a droplet of water on the nanofiber mats collected on a glass slide. The other method was to place a piece of electrospun hybrid film on wet paper. All these processes were recorded using a digital camera (PowerShot SX50HS, Canon, Tokyo, Japan) [76,94].

## 3. Results and Discussion

### 3.1. Electrospinning Is Explored to Transfer LQK into Nanofiber Mats

**Figure 1:**
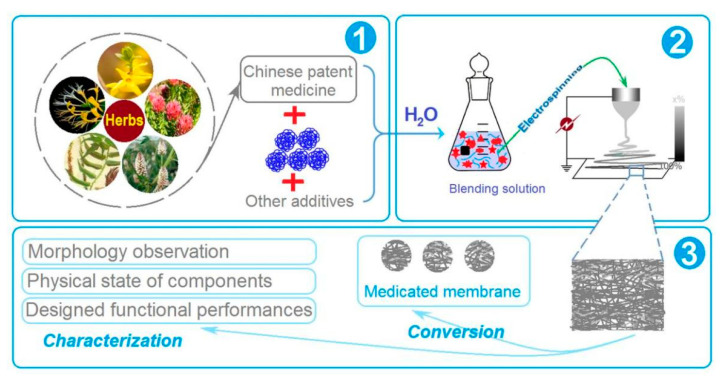
A scheme showing the procedure from the (**1**) reasonable selection of matched raw materials, to (**2**) facile implementation of a green preparation using handheld electrospinning, and to (**3**) systematic characterizations of the nanofibers and the converted medicated membranes.

In the market, LQK has two traditional dosage forms, one is in particles and the other is in capsules. The active ingredients are extracted from herbs including forsythia, honeysuckle, prepared ephedra, fried bitter almond, gypsum, isatidis root, cotton horse rhizome, houttuynia, patchouli, rhubarb, rhodiola, menthol and licorice [91]. The LQK is brownish yellow to brown particles, with gas that is slightly fragrant and tastes slightly bitter. In this work, a strategy is developed for transferring these particles into medicated hybrid films, which is exhibited in Figure 1. A filament-forming polymer—PVP, and a common sweeter sucralose were co-dissolved with LQK into water to prepare the electrospinnable working fluids. PVP is a well-known filament-forming polymeric matrix and also a useful solubility enhancer of a wide variety of poorly water-soluble drugs. Thus, the combinations of PVP, sucralose and LQK are anticipated to promote the fast dissolution of active ingredients from the herb extracts and meanwhile change them to a favorable taste for the patients. Accordingly, the handheld electrospinning is a green process healthy to the environment and also benefits the final products, thanks to being free of any organic solvents. Finally, the resultant nanofiber mats could be cut into circular films for easy oral administration.

Electrospinning is a special micro- and nanofiber manufacturing process in which a polymer solution is jet-spun in a strong electric field [68,69,70,71,72], just as its EHDA brother method, electrospraying [95,96,97]. Under the action of the applied high voltage, the droplets at the nozzle are changed from a spherical to a conical shape (i.e., the famous Taylor cone), and later split and stretched from the tip of the cone to remove the loaded solvents for creating solid micro- and nanodiameter fibers. In the HHE-1 handheld electrospinning equipment, two cells are utilized to provide the high voltage (typically 0~10 ± 1 kV, and the rated current is often within 90 mA). Its whole weight is only 133 g, very slight for application. The designed syringe size is 5 mL. The suitable working ambient conditions are similar to other electrospinning systems, i.e., a temperature range of 0~40 °C and a working environment relative humidity of smaller than 80%.

**Figure 2:**
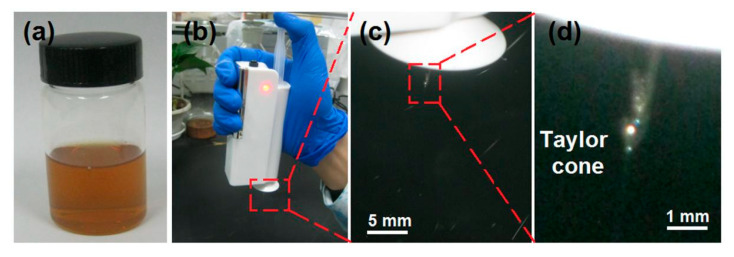
The handheld electrospinning apparatus and the typical electrospinning processes: (**a**) the brown color co-dissolved working fluid; (**b**) the operation of the handheld electrospinning apparatus; (**c**,**d**) the electrospinning working processes and the typical Taylor cone.

Shown in Figure 2a is the working fluid, which has a brown color. The HHE-1 handheld electrospinning instrument is flexible and easy to operate. A syringe (5 mL) was perfused with the prepared working fluid, and then put into the body of the HHE-1 handheld instrument, a stainless-steel needle (20 G × 38 mm length) was connected with the syringe, and later, the button was pushed to start the machine. When the red indicator light was always on (Figure 2b), the operator held the instrument to keep a certain distance (here 15 cm) from the receiving aluminum foil. The operator needed to keep his press continuously on the button of the electrospinning apparatus, and slowly push the pushing rod of the syringe with the thumb. After adjusting the distance between the apparatus and the receiving foil and the speed with which the thumb pushed the pushing rod to achieve the best effect, the nanofiber mats could be collected. For safe operation, it is strictly forbidden to touch the needle and the directional cover when the instrument is powered on, so as to avoid the danger of applied high voltage. In the present study, a typical electrospinning process and the typical Taylor cone are given in Figure 2c,d, respectively.

### 3.2. The Morphologies of the LQK-Loaded Nanofibers and Their Transferring to Orodispersible Films

**Figure 3:**
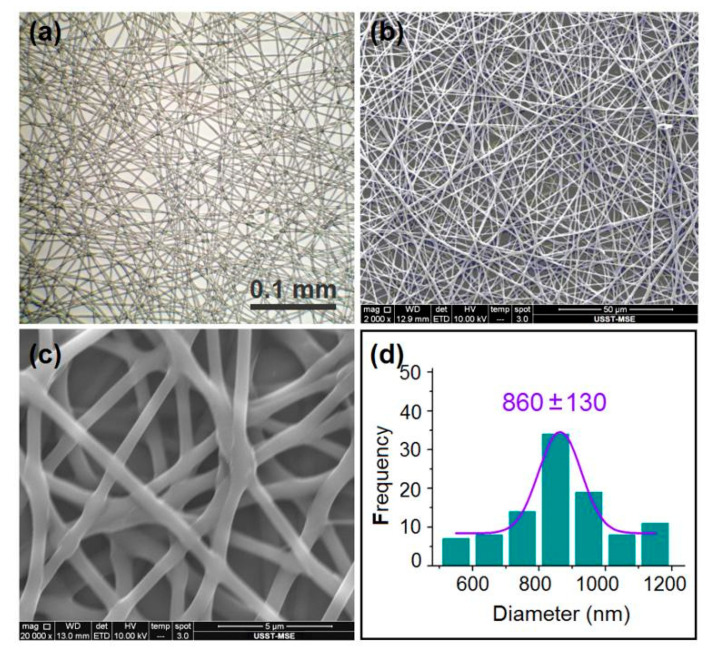
Optical images (**a**), SEM images (**b**,**c**) and the size distributions of the prepared nanofibers (**d**).

The morphologies of the electrospun nanofibers detected using different microscopes are shown in Figure 3. Both the OM (Figure 3a) and SEM images (Figure 3b,c) indicate that these nanofibers presented in a straight linear format, with few discerned beads-on-a-string or spindles-on-a-string phenomena. They have an estimated diameter of 860 ± 130 nm (Figure 3d). As a linear polymer, PVP has fine filament-forming properties. Meanwhile, PVP has a fine solubility in water and a wide variety of organic solvents such as ethanol, methanol and acetone. Thus, PVP, being authorized by the Food and Drug Administration (FDA) for biomedical applications, is frequently explored for applications in many sorts of pharmaceutical dosage forms such as pellets, tablets and capsules. Here, the LQK-loaded films demonstrate an additional manner for modernizing traditional Chinese patent medicines using PVP as a polymeric matrix.

**Figure 4:**
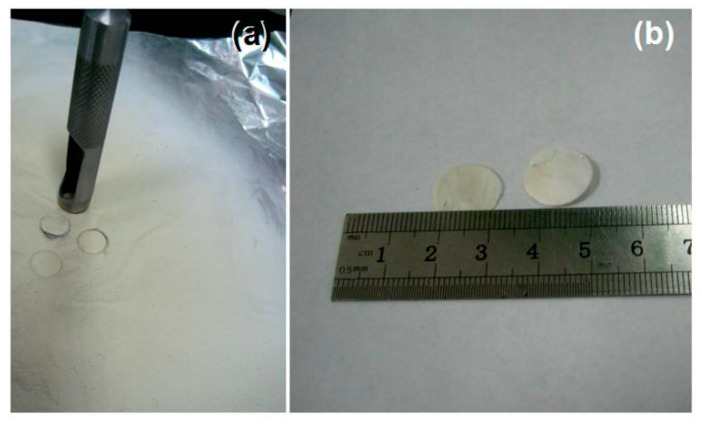
Conversion of the electrospun nanofiber mats to orodispersible films: (**a**) puncher is exploited to cut membrane; (**b**) the diameter of the cut circle.

Electrospun medicated nanofibers are mainly intermediate dosage forms, which can be transferred into different final dosage forms for various administration applications [93]. Here, a puncher was used to transfer the electrospun nanofiber mats into orodispersible films. The digital pictures in Figure 4 show the whole process. The stainless-steel puncher has a circle cut diameter of 15 mm. After pressing on the electrospun nanofiber mats with force (Figure 4a), a circle with a diameter of 15 mm (Figure 4b) can be cut off for administration applications. The weight of each film is 7.8 ± 1.4 mg (*n* = 10), and thus containing 2.5 ± 0.4 mg LQK.

### 3.3. Physical State of the Components Loaded into the Films

**Figure 5:**
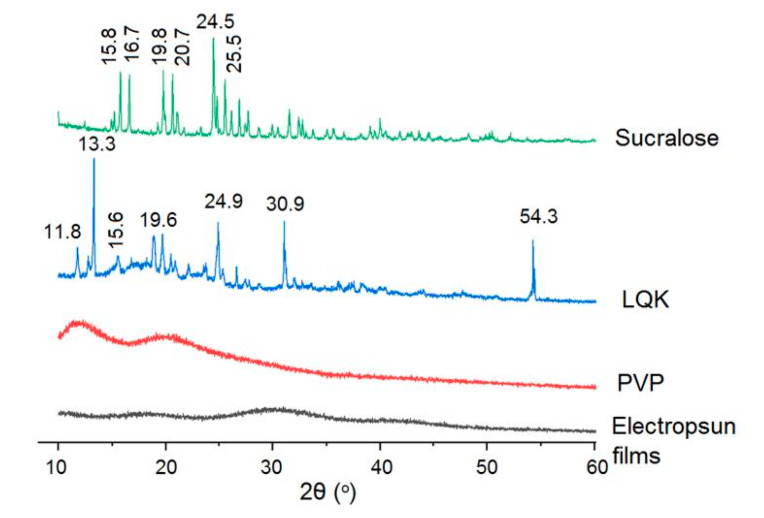
XRD patterns of the raw materials sucralose, LQK, PVP and their electrospun films.

The crystal property of the raw materials and the electrospun nanofibers can be detected using the XRD patterns. Shown in Figure 5, it is clear that sucralose presents in a crystalline format and the granular mixtures of LQK contain some crystal active ingredients. PVP is a well-known amorphous linear polymer, which has fine electrospinnability and is able to prevent the crystallization of many drugs. As anticipated, the electrospun films were also in an amorphous state. The crystalline materials in LQK and sucralose lost the crystal state and were converted into the hybrid films amorphously during the electrospinning processes, which could benefit the fast dissolution of LQK’s ingredients due to no lattice energy needing to be overcome for dissolution.

**Figure 6:**
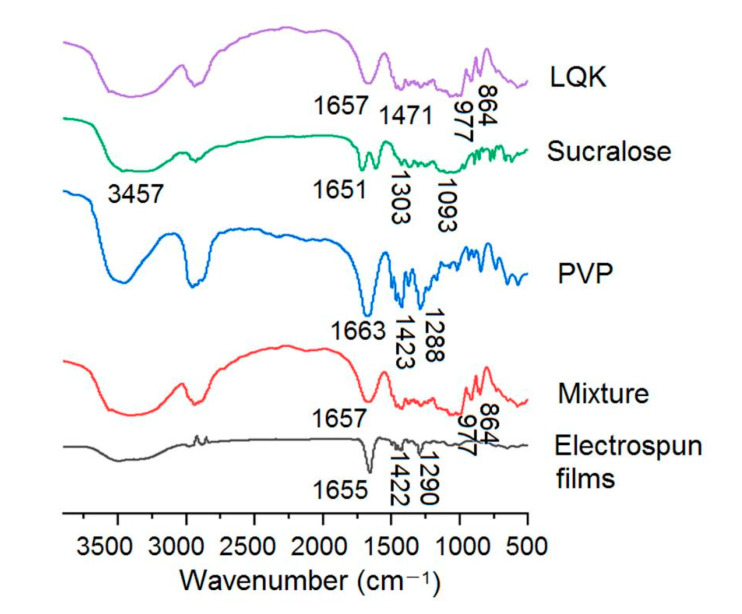
FTIR spectra of the raw materials sucralose, LQK, PVP and their electrospun films.

Shown in Figure 6 are spectra of the raw materials (LQK, sucralose and PVP) and their electrospun nanofibers. A physical mixture of the raw materials was detected for comparison. LQK is a mixture containing a series of active herb extracts. It has characteristic peaks at 1657, 1471, 977 and 864 cm^−1^. These peaks can also be found in the spectra of the mixture. However, in the spectra of electrospun films, they disappeared. These phenomena suggest that the favorite secondary interactions should happen between those active herb extracts and PVP, which maybe include hydrogen bonding, hydrophobic interaction and electrostatic interactions [28,46,76]. This is favorable, on the one hand, for the fast dissolution of LQK. On the other hand, it is desirable for the stable storage of the films.

### 3.4. The Fast-Disintegrating Performances of the LQK-Loaded Films

**Figure 7:**
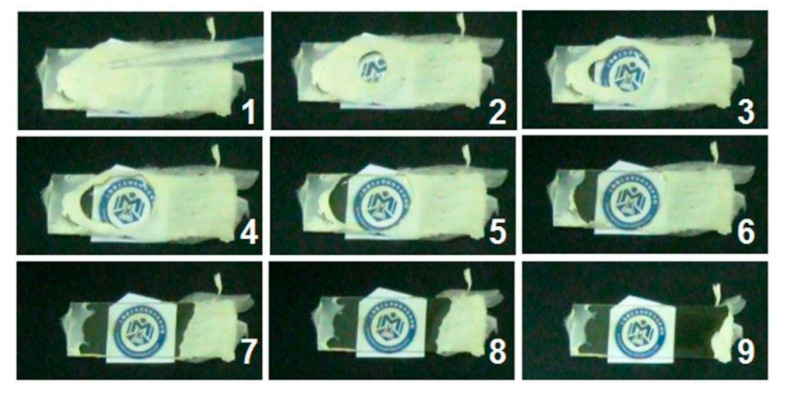
A direct showing of the fast dissolution of the electrospun medicated films.

As shown in Figure 7, when a drop of water was dripped onto the slide glass covered with the electrospun nanofiber mats, the logo of “School of Materials and Chemistry” appeared gradually but quickly. The time period from “1” to “9” was just 6 s, suggesting the easy and rapid dissolution of PVP, sucralose and the loaded active herb extracts in LQK.

**Figure 8:**
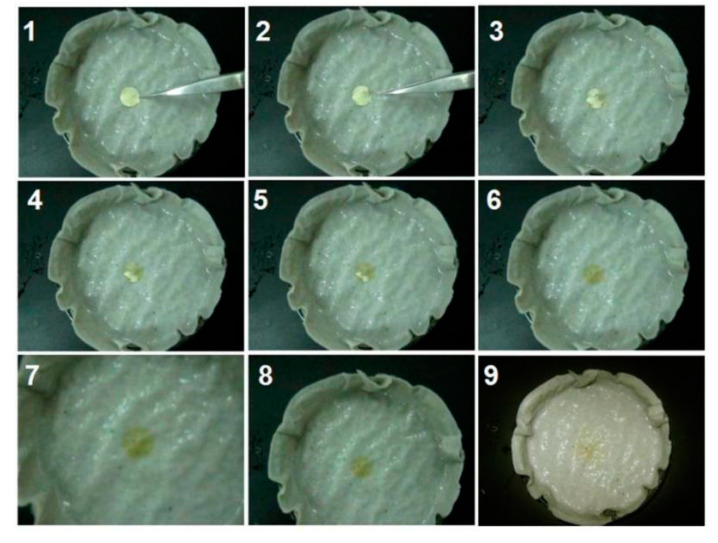
Fast-disintegrating experiments of an orodispersible film on the interfacial tongue.

Two papers were superposed and placed on a Petri dish. After wetting with some water to mimic a tongue, a piece of the cut film was placed on the wet paper. A camera was used to record the whole process. The dissolution and passive diffusion processes were shown in Figure 8. The time from “1” to “6”, which is a water-absorbing and gelling process, took only 4.2 ± 0.6 s. After absorbing water, the opaque and slight yellow color of the membrane was gradually turned to a yellow-brown color and transparent. Apparently, the hygroscopicity and hydrophilicity of the PVP matrix, the small diameter of nanofibers, the amorphous state of the components disclosed by XRD patterns and the three-dimensional web structure of the nanofiber films acted together to promote the gelling processes. Later, the yellow-brown color was gradually weakened, as shown from “7” to “9”. This is a passive diffusion process, lasting a relatively longer time period of 91.7 ± 11.4 s. If extra stirring was added (such as mimicking the tongue movement), the diffusion and transportation processes should be still very quick. Incidentally, the films need to be stored in an environment with a low humidity due to the hygroscopicity of the polymeric carrier, i.e., PVP, which is also a common issue for numerous traditional dosage forms.

## 4. Conclusions and Perspectives

With PVP K60 as a drug carrier and also a filament-forming polymeric matrix, a handheld electrospinning apparatus was exploited to prepare hybrid films containing active herb extracts in a traditional Chinese patent medicine, LQK. A sweeter sucralose was co-loaded into the working fluids, which can be explored to cover up the bitter taste of the original commercial LQK products. The electrospun LQK-loaded films were demonstrated to be linear nanofibers, which had a diameter of 860 ± 140 nm. A puncher was utilized to transfer the electrospun mats into circular films. XRD and FTIR experiments demonstrated that the green electrospinning process successfully converted the crystalline herb components and sucralose into an amorphous state due to the secondary interactions. Two self-created experiments verified that the prepared orodispersible films showed the desired functional performance in promoting the fast disintegration and dissolution of LQK, which can increase the convenience for the patients. The sweet taste and the convenience of administration should improve the patients’ compliance of LQK.

The present study pioneers a concept for the modernization of traditional Chinese medicines and for effectively delivering herb extracts. Often these medicines and herb extracts are known as safe for the human body due to a natural source of herbs. However, their effectiveness and convenience need to be improved. The electrospun polymeric nanocomposite membrane can be a useful strategy for transferring them into a more efficacious dosage form. Particularly, the liquid and paste dosage forms are very frequently in traditional Chinese patent medicines due to an extraction process of the active ingredients from the herbs. These dosage forms can be solidified into solid nanofibers for easy shipping and transportation, and also a longer time period of the stability of active ingredients. Additionally, the combined therapy of traditional Chinese medicine and Western medicine is popular. The present protocols can be further extended to prepare dosage forms that contain both the Chinese patent medicines and bioactive chemical little molecules in future.

## Data Availability

The data supporting the findings of this manuscript are available from the corresponding authors upon reasonable request.

## Abbreviations

Coronavirus Disease 2019: COVID-19; Lianhua Qingwen Keli: LQK; Electrohydrodynamic atomization: EHDA; Polyvinylpyrrolidone K60: PVP K 60; Scanning electron microscope: SEM; Field-emission scanning electron microscope: FESEM; X-ray diffraction: XRD; Fourier transform infrared: FTIR; Food and Drug Administration: FDA.

## References

[1] Tubtimsri S., Weerapol Y. (2021). Improvement in solubility and absorption of nifedipine using solid solution: Correlations beween surface free energy and drug dissolution. Polymers.

[2] Leonarta F., Lee C.K. (2021). Nanofibrous membrane with encapsulated glucose oxidase for self-sustained antimicrobial applications. Membranes.

[3] Ding C.B., Zhou C.X., Fan Y.Y., Liu Q., Zhang H.F., Wu Z.W. (2022). Electrospun polylactic acid/sulfadiazine sodium/proteinase nanofibers and their applications in treating frostbite. J. Appl. Polym. Sci..

[4] Liu X., Xu H., Zhang M., Yu D.G. (2021). Electrospun medicated nanofibers for wound healing—Review. Membranes.

[5] Zare M., Dziemidowicz K., Williams G.R., Ramakrishna S. (2021). Encapsulation of pharmaceutical and nutraceutical active ingredients using electrospinning processes. Nanomaterials.

[6] Peres R.M., Sousa J.M.L., Oliveira M.O.D., Rossi M.V., Oliveira R.R.D., Lima N.B.D., Bernussi A., Warzywoda J., Sarmento B., Munhoz A.H. (2021). Pseudoboehmite as a drug delivery system for acyclovir. Sci. Rep..

[7] Mehta P., Rasekh M., Patel M., Onaiwu E., Nazari K., Kucuk I., Wilson P.B., Arshad M.S., Ahmad Z., Chang M.W. (2021). Recent applications of electrical, centrifugal, and pressurised emerging technologies for fibrous structure engineering in drug delivery, regenerative medicine and theranostics. Adv. Drug Deliv. Rev..

[8] Cervantes M.Y.G., Kim J., Chitara B., Wymer N., Yan F. (2021). N-halamine-decorated electrospun polyacrylonitrile nanofibrous membranes: Characterization and antimicrobial properties. React. Funct. Polym..

[9] Kumar D., Kumar S., Kumar S., Rohatgi S., Kundu P.P. (2021). Synthesis of rifaximin loaded chitosan-alginate core-shell nanoparticles (Rif@CS/Alg-NPs) for antibacterial applications. Int. J. Biol. Macromol..

[10] Wlodarczyk J., Stojko M., Musial-Kulik M., Karpeta-Jarzabek P., Pastusiak M., Janeczek H., Dobrzynski P., Sobota M., Kasperczyk J. (2022). Dual-jet electrospun PDLGA/PCU nonwovens and their mechanical and hydrolytic degradation properties. J. Mech. Behav. Biomed..

[11] Yu D.G. (2021). Preface-bettering drug delivery knowledge from pharmaceutical techniques and excipients. Curr. Drug Deliv..

[12] Bae Y., Kim Y., Lee E.S. (2021). Endosomal pH-responsive Fe-based hyaluronate nanoparticles for doxorubicin delivery. Molecules.

[13] Hajjari M.M., Golmakani M.T., Sharif N., Niakousari M. (2021). In-vitro and in-silico characterization of zein fiber incorporating cuminaldehyde. Food Bioprod. Process..

[14] Ullah A., Saito Y., Ullah S., Haider M.K., Nawaz H., Duy-Nam P., Kharaghani D., SooKim I. (2021). Bioactive sambong oil-loaded electrospun cellulose acetate nanofibers: Preparation, characterization, and in-vitro biocompatibility. Int. J. Biol. Macromol..

[15] Feng X.C., Hao J.S. (2021). Identifying new pathways and targets for wound healing and therapeutics from natural sources. Curr. Drug Deliv..

[16] Vineis C., Maya I.C., Mowafi S., Varesano A., Ramírez D.O.S., Taleb M.A., Tonetti C., Guarino V., El-Sayed H. (2021). Synergistic effect of sericin and keratin in gelatin based nanofibers for in vitro applications. Int. J. Biol. Macromol..

[17] Bagliotti M.A., Miguel S.R., Chaves D.S.M.P., Perosa F.R., Gomes D.O.A., Marlus C. (2021). Cellulose nanofibers improve the performance of retrograded starch/pectin microparticles for colon-specific delivery of 5-ASA. Pharmaceutics.

[18] Song Y., Huang H., He D., Yang M., Wang H., Zhang H., Li J., Li Y., Wang C. (2021). Gallic acid/2-hydroxypropyl-β-cyclodextrin inclusion complexes electrospun nanofibrous webs: Fast dissolution, improved aqueous solubility and antioxidant property of gallic acid. Chem. Res. Chin. Univ..

[19] Saraogi G.K., Tholiya S., Mishra Y., Mishra V., Albuttim A., Nayak P., Tambuwala M.M. (2022). Formulation development and evaluation of pravastatin-loaded nanogel for hyperlipidemia management. Gels.

[20] Naidoo S., Daniels A., Habib S., Singh M. (2022). Poly-l-lysine–lactobionic acid-capped selenium nanoparticles for liver-targeted gene delivery. Int. J. Mol. Sci..

[21] Hou Z., Itagaki N., Kobayashi H., Tanaka K., Takarada W., Kikutani T., Takasaki M. (2021). Bamboo charcoal/poly(l-lactide) fiber webs prepared using laser-heated melt electrospinning. Polymers.

[22] Hardt J.C., Pellá M.C.G., Meira A.C.R., Rosenberger A.G., Caetano J., Dragunski D.C. (2021). Potential wound dressings from electrospun medicated poly(butylene-adipate-co-terephthalate)/poly-(ε-caprolactone) microfibers. J. Mol. Liq..

[23] Vu Q.M., Nguyen T.C., Dam D.M.N., Vu Q.T., Le T.L., Hoang T.D., Tran T.K.N., Nguyen T.A., Nguyen P.H., Thai H. (2021). A novel method for preparation of carrageenan/fish scale collagen/allopurinol biocomposite film. Int. J. Polym. Sci..

[24] Łyszczarz E., Brniak W., Szafraniec-Szczęsny J., Majka T.M., Majda D., Zych M., Pielichowski K., Jachowicz R. (2021). The impact of the preparation method on the properties of orodispersible films with aripiprazole: Electrospinning vs. casting and 3D printing methods. Pharmaceutics.

[25] Sa’adon S., Ansari M.N.M., Razak S.I.A., Anand J.S., Nayan N.H.M., Ismail A.E., Khan M.U.A., Haider A. (2021). Preparation and physicochemical characterization of a diclofenac sodium-dual layer polyvinyl alcohol patch. Polymers.

[26] Enikov R.E.T., Anton R. (2021). Method for production of aligned nanofibers and fiber elasticity measurement. J. Mech. Behav. Biomed. Mater..

[27] Liu Y., Chen X., Liu Y., Gao Y., Liu P. (2022). Electrospun coaxial fibers to optimize the release of poorly water-soluble drug. Polymers.

[28] Liu Y., Chen X., Yu D.G., Liu H., Liu Y., Liu P. (2021). Electrospun PVP-core/PHBV-shell nanofibers to eliminate tailing off for an improved sustained release of curcumin. Mol. Pharm..

[29] Yu D.G., Lv H. (2022). Preface-striding into nano drug delivery. Curr. Drug. Deliv..

[30] Kiss K., Hegedüs K., Vass P., Vári-Mező D., Farkas A., Nagy Z.K., Molnár L., Tóvári J., Mező G., Marosi G. (2021). Development of fast-dissolving dosage forms of curcuminoids by electrospinning for potential tumor therapy application. Int. J. Pharm..

[31] Zhang Y., Li S., Xu Y., Shi X., Zhang M., Huang Y. (2022). Engineering of hollow polymeric nanosphere-supported imidazolium-based ionic liquids with enhanced antimicrobial activities. Nano Res..

[32] Lukiev I.V., Antipina L.S., Goreninskii S.I., Tverdokhlebova T.S., Vasilchenko D.V., Nemoykina A.L., Goncharova D.A., Svetlichnyi V.A., Dambaev G.T., Bouznik V.M. (2021). Antibacterial ferroelectric hybrid membranes fabricated via electrospinning for wound healing. Membranes.

[33] Zhang L., Li L.F., Wang L.C., Nie J., Ma G.P. (2020). Multilayer electrospun nanofibrous membranes with antibacterial property for air filtration. Appl. Surf. Sci..

[34] Salim S.A., Kamoun E.A., Evans S., EL-Moslamy S.H., El-Fakharany E.M., Elmazar M.M., Abdel-Aziz A.F., Abou-Saleh R.H., Salaheldin T.A. (2021). Mercaptopurine-loaded sandwiched tri-layered composed of electrospun polycaprolactone/poly(methyl methacrylate) nanofibrous scaffolds as anticancer carrier with antimicrobial and antibiotic features: Sandwich configuration nanofibers, release study and in vitro bioevaluation tests. Int. J. Nanomed..

[35] Manakhov A.M., Sitnikova N.A., Tsygankova A.R., Alekseev A.Y., Adamenko L.S., Permyakova E., Baidyshev V.S., Popov Z.I., Blahová L., Eliáš M. (2021). Electrospun biodegradable nanofibers coated homogenously by Cu magnetron sputtering exhibit fast ion release. Computational and experimental study. Membranes.

[36] Mirzaie Z., Reisi-Vanani A., Barati M., Atyabi S.M. (2021). The drug release kinetics and anticancer activity of the GO/PVA-curcumin nanostructures: The effects of the preparation method and the GO amount. J. Pharm. Sci..

[37] Zhou K.C., Wang M.L., Zhou Y.Q., Sun M.J., Xie Y.F., Yu D.G. (2022). Comparisons of antibacterial performances between electrospun polymer@drug nanohybrids with drug-polymer films. Adv. Compos. Hybrid Mater..

[38] Aljohani M., Alkabli J., Abualnaja M.M., Alrefaei A.F., Almehmadi S.J., Mahmoud M.H.H., El-Metwaly N.M. (2021). Electrospun AgNPs-polylactate nanofibers and their antimicrobial applications. React. Funct. Polym..

[39] Wu C., Wei X.H., Zhao K., Jiang J.Y., Jiao M.Z., Cheng J.H., Tang Z., Guo Z., Tang Y.F. (2021). Novel dam-like effect based on piezoelectric energy conversion for drug sustained release of drug-loaded TiO_2_ @ BaTiO_3_ coaxial nanotube coating. Ceram. Int..

[40] Wable V., Biswas P.K., Moheimani R., Aliahmad N., Omole P., Siegel A.P., Agarwal M., Dalir H. (2021). Engineering the electrospinning of MWCNTs/epoxy nanofiber scaffolds to enhance physical and mechanical properties of CFRPs. Compos. Sci. Technol..

[41] Karki S., Kim H., Na S.-J., Shin D., Jo K., Lee J. (2016). Thin films as an emerging platform for drug delivery. Asian J. Pharm. Sci..

[42] Zhang X., Yao D., Zhao W.Y., Zhang R., Yu B.R., Ma G.P., Li Y., Hao D.F., Xu F.J. (2021). Engineering platelet-rich plasma based dual-network hydrogel as a bioactive wound dressing with potential clinical translational value. Adv. Funct. Mater..

[43] Haidar M.K., Timur S.S., Demirbolat G.M., Nemutlu E., Gürsoy R.N., Ulubayram K., Öner L., Eroğlu H. (2021). Electrospun nanofibers for dual and local delivery of neuroprotective drugs. Fiber. Polym..

[44] Mouro C., Fangueiro R., Gouveia I.C. (2020). Preparation and characterization of electrospun double-layered films membranes as a carrier for *Centella asiatica* (L.). Polymers.

[45] Zhang M.X., Song W.L., Tang Y.X., Xu X.Z., Huang Y.N., Yu D.G. (2022). Polymer-based nanofifiber-nanoparticle hybrids and their medical applications. Polymers.

[46] He H., Wu M., Zhu J.W., Yang Y.Y., Ge R.L., Yu D.G. (2021). Engineered spindles of little molecules around electrospun nanofibers for biphasic drug release. Adv. Fiber Mater..

[47] Lee B., Song Y., Park C., Kim J., Kang J., Lee H., Yoon J., Cho S. (2021). Focused patterning of electrospun nanofibers using a dielectric guide structure. Polymers.

[48] Li Y., Wang D., Xu G.C., Qiao L., Li Y., Gong H.Y., Shi L., Li D.W., Gao M., Liu G.R. (2021). ZIF-8/PI nanofibrous membranes with high-temperature resistance for highly efficient PM0.3 air filtration and oil-water separation. Front. Chem..

[49] Kazsoki A., Palcsó B., Alpár A., Snoeck R., Andrei G., Zelkó R. (2022). Formulation of acyclovir (core)-dexpanthenol (sheath) nanofibrous patches for the treatment of herpes labialis. Int. J. Pharm..

[50] Chi Z.M., Zhao S.Q., Feng Y.X., Yang L. (2020). On-line dissolution analysis of multiple drugs encapsulated in electrospun nanofibers. Int. J. Pharm..

[51] Chen J., Zhang G., Zhao Y., Zhou M., Zhong A., Sun J. (2022). Promotion of skin regeneration through co-axial electrospun fibers loaded with basic fibroblast growth factor. Adv. Compos. Hybrid Mater..

[52] Kalous T., Holec P., Erben J., Bilek M., Batka O., Pokorny P., Chaloupek J., Chvojka J. (2021). The optimization of alternating current electrospun PA 6 solutions using a visual analysis system. Polymers.

[53] Wang L.Y., Cheng W., Zhu J.J., Li W.Y., Li D.Y., Yang X., Zhao W.X., Ren M.J., Ren J.J., Mo X.M. (2022). Electrospun nanoyarn and exosomes of adipose-derived stem cells for urethral regeneration: Evaluations in vitro and in vivo. Colloid Surf. B Biointerface.

[54] Terra A.L.M., Moreira J.B., Costa J.A.V., Morais M.G.D. (2021). Development of time-pH indicator nanofibers from natural pigments: An emerging processing technology to monitor the quality of foods. LWT.

[55] Li J.K., Guan S.M., Su J.J., Liang J.H., Cui L.L., Zhang K. (2020). The Development of hyaluronic acids used for skin tissue regeneration. Curr. Drug Deliv..

[56] Sahoo S.K., Panigrahi G.K., Sahoo J.K., Pradhan A.K., Purohit A.K., Dhal J.P. (2021). Electrospun magnetic polyacrylonitrile-GO hybrid nanofibers for removing Cr(VI) from water. J. Mol. Liq..

[57] Wang K.L., Wang X.Y., Jiang D., Pei Y.F., Wang Z., Zhou X.J., Wu J.L., Mo X.M., Wang H.S. (2022). Delivery of mRNA vaccines and anti-PDL1 siRNA through non-invasive transcutaneous route effectively inhibits tumor growth. Compos. Part B-Eng..

[58] Yuan Z.C., Sheng D.D., Jiang L.P., Shafiq M., Khan A.U.R., Hashim R., Chen Y.J., Li B.J., Xie X.R., Chen J. (2022). Vascular Endothelial Growth Factor-Capturing Aligned Electrospun Polycaprolactone/Gelatin Nanofibers Promote Patellar Ligament Regeneration. Acta Biomater..

[59] Song X.R., Jiang Y.X., Zhang W.X., Elfawal G., Wang K.L., Jiang D., Hong H.Y., Wu J.L., He C.L., Mo X.M. (2022). Transcutaneous tumor vaccination combined with anti-programmed death-1 monoclonal antibody treatment produces a synergistic antitumor effect. Acta Biomater..

[60] Darbasizadeh B., Mortazavi S.A., Kobarfard F., Jaafari M.R., Hashemi A., Farhadnejad H., Feyzi-barnaji B. (2021). Electrospun doxorubicin-loaded PEO/PCL core/sheath nanofibers for chemopreventive action against breast cancer cells. J. Drug. Deliv. Sci. Technol..

[61] Sivan M., Madheswaran D., Valtera J., Kostakova E.K., Lukas D. (2022). Alternating current electrospinning: The impacts of various high-voltage signal shapes and frequencies on the spinnability and productivity of polycaprolactone nanofibers. Mater. Des..

[62] Kang S.X., Hou S.C., Chen X.W., Yu D.G., Wang L., Li X.Y., Williams G.R. (2020). Energy-saving electrospinning with a concentric Teflon-core rod spinneret to create medicated nanofibers. Polymers.

[63] Mehdi M., Hussain S., Gao B.B., Shah K.A., Mahar F.K., Yousif M., Hussain S., Ahmed F. (2021). Fabrication and characterization of rizatriptan loaded pullulan nanofibers as oral fast-dissolving drug system. Mater. Res. Express..

[64] Ebhodaghe S.O. (2022). A scoping review on the biomedical applications of polymeric particles. Int. J. Polym. Mater. Polym. Biomater..

[65] Bukhary H., Williams G.R., Orlu M. (2020). Fabrication of electrospun levodopa-carbidopa fixed-dose combinations. Adv. Fiber Mater..

[66] Miranda C.S., Silva A.F.G., Pereira-Lima S.M.M.A., Costa S.P.G., Homem N.C., Felgueiras H.P. (2022). Tunable spun fiber constructs in biomedicine: Influence of processing parameters in the fibers’ architecture. Pharmaceutics.

[67] Ismail I., Bakar N.F.A., Tan H.L., Ideris N., Zain Z.M., Idris S.S., Radacsi N. (2021). Ultra-sensitive electrosprayed AuNPs-decorated PAA/PAN electrospun nanofibers as glucose sensor. J. Mater. Res..

[68] Zhang X., Guo S., Qin Y., Li C. (2021). Functional electrospun films for efficient oxygen reduction reaction. Chem. Res. Chin. Univ..

[69] Kang S., Zhao K., Yu D.G., Zheng X., Huang C. (2022). Advances in biosensing and environmental monitoring based on electrospun nanofibers. Adv. Fiber Mater..

[70] El-Shanshory A.A., Agwa M.M., Abd-Elhamid A.I., Soliman H.M.A., Mo X.M., Kenawy E.R. (2022). Metronidazole Topically Immobilized Electrospun Nanofibrous Scaffold: Novel Secondary Intention Wound Healing Accelerator. Polymers.

[71] Bhusnure O.G., Gholve S.B., Giram P.S., Gaikwad A.V., Udumansha U., Mani G., Tae J.H. (2021). Novel 5-flurouracil-Embedded non-woven PVA-PVP electrospun nanofibers with enhanced anti-cancer efficacy: Formulation, evaluation and in vitro anti-cancer activity. J. Drug. Deliv. Sci. Technol..

[72] Brimo N., Serdaroğlu D. (2021). Çökeliler; Uysal, B. Comparing Antibiotic Pastes with Electrospun Nanofibers as Modern Drug Delivery Systems for Regenerative Endodontics. Curr. Drug Deliv..

[73] Xu X.Z., Zhang M.X., Lv H., Yang Y.Y., Yu D.G. (2022). Electrospun polyacrylonitrile-based lace nanostructures and their Cu(II) adsorption. Sep. Purif. Technol..

[74] Malihe G., Shahnoosh A., Amir R., Shiva R., Hajir B.S. (2022). Fabrication and characterization of chitosan-polycaprolactone core-shell nanofibers containing tetracycline hydrochloride. Colloid Surf. A Physicochem. Eng. Asp..

[75] Zhang Z., Zhao Y., Li Z., Zhang L., Liu Z., Long Z., Li Y., Liu Y., Fan R., Sun K. (2021). Synthesis of carbon/SiO_2_ core-sheath nanofibers with Co-Fe nanoparticles embedded in via electrospinning for high-performance microwave absorption. Adv. Compos. Hybrid Mater..

[76] Ning T., Zhou Y., Xu H.X., Guo S., Yu D.G. (2021). Orodispersible membranes from a modified coaxial electrospinning for fast dissolution of diclofenac sodium. Membranes.

[77] Huang C., Dong J., Zhang Y., Chai S., Wang X., Kang S., Yu D., Wang P., Jiang Q. (2021). Gold Nanoparticles-Loaded Polyvinylpyrrolidone/Ethylcellulose Coaxial Electrospun Nanofibers with Enhanced Osteogenic Capability for Bone Tissue Regeneration. Mater. Des..

[78] Abdullah M.F., Andriyana A., Muhamad F., Ang B.C. (2021). Effect of core-to-shell flowrate ratio on morphology, crystallinity, mechanical properties and wettability of poly(lactic acid) fibers prepared via modified coaxial electrospinning. Polymer.

[79] Zhao K., Lu Z.H., Zhao P., Kang S.X., Yang Y.Y., Yu D.G. (2021). Modified tri-axial electrospun functional core–shell nanofibrous membranes for natural photodegradation of antibiotics. Chem. Eng. J..

[80] Ghosal K., Augustine R., Zaszczynska A., Barman M., Jain A., Hasan A., Kalarikkal N., Sajkiewicz P., Thomas S. (2021). Novel drug delivery systems based on triaxial electrospinning based nanofibers. React. Funct. Polym..

[81] Wang M., Tan Y., Li D., Xu G., Yin D., Xiao Y., Xu T., Chen X., Zhu X., Shi X. (2021). Negative isolation of circulating tumor cells using a microfluidic platform integrated with streptavidin-functionalized PLGA nanofibers. Adv. Fiber Mater..

[82] Wang Y.B., Xu H.X., Wu M., Yu D.G. (2021). Nanofibers-Based Food Packaging. ES Food Agrofor..

[83] Qi H.N., Wang G.Y., Ma Q.L., Li D., Dong X.T., Yu W.S., Wang J.X., Liu G.X., Zhang X.J. (2022). Conjugative electrospinning towards Janus-type nanofibers array membrane concurrently displaying dual-functionality of improved red luminescence and tuneable superparamagnetism. J. Mater. Sci. Mater. Electron..

[84] Qi H.N., Xie Y.R., Yang L., Tang X.H., Ma Q.L., Yu W.S., Dong X.T., Li D., Liu G.X., Wang J.X. (2021). Electrospun polyfunctional switch-typed anisotropic photoconductive film endued with superparamagnetic-fluorescent performances. Appl. Mater. Today.

[85] Yu D.G., Wang M.L., Ge R. (2021). Strategies for sustained drug release from electrospun multi-layer nanostructures. Wiley Interdiscip. Rev. Nanomed. Nanobiotechnol..

[86] Wang M.L., Hou J.S., Yu D.G., Li S.Y., Zhu J.W., Chen Z.Z. (2020). Electrospun tri-layer nanodepots for sustained release of acyclovir. J. Alloys Compd..

[87] Lee J., Moon S., Han Y.B., Yang S.J., Lahann J., Lee K.J. (2021). Facile fabrication of anisotropic multicompartmental microfibers using charge reversal electrohydrodynamic co-jetting. Macromol. Rapid Commun..

[88] Kamath S.M., Sridhar K., Jaison D., Gopinath V., Ibrahim B.K.M., Gupta N., Sundaram A., Sivaperumal P., Padmapriya S., Patil S.S. (2020). Fabrication of tri-layered electrospun polycaprolactone mats with improved sustained drug release profile. Sci. Rep..

[89] Liu Y.J., Wang J.L., Shao Y., Deng R.H., Zhu J.T., Yang Z.Z. (2022). Recent advances in scalable synthesis and performance of Janus polymer/inorganic films. Prog. Mater. Sci..

[90] Bertani R., Bartolozzi A., Pontefisso A., Quaresimin M., Zappalorto M. (2021). Improving the antimicrobial and mechanical properties of epoxy resins via nanomodification: An Overview. Molecules.

[91] (2021). Drug Administration Instruction: Lianhua Qingwen Keli.

[92] Lv H., Guo S., Zhang G., He W., Wu Y., Yu D.-G. (2021). Electrospun structural hybrids of acyclovir-polyacrylonitrile at acyclovir for modifying drug release. Polymers.

[93] Vass P., Szabó E., Domokos A., Hirsch E., Galata D., Farkas B., Démuth B., Andersen S.K., Vigh T., Verreck G. (2020). Scale-up of electrospinning technology: Applications in the ph armaceutical industry. Wiley Interdiscip. Rev. Nanomed. Nanbiotechnol..

[94] Khalid G.M., Selmin F., Musazzi U.M., Gennari C.G.M., Minghetti P., Cilurzo F. (2021). Trends in the characterization methods of orodispersible films. Curr. Drug Deliv..

[95] Moreira A., Lawson D., Onyekuru L., Dziemidowicz K., Angkawinitwong U., Costa P.F., Radacsi N., Williams G.R. (2021). Protein encapsulation by electrospinning and electrospraying. J. Control. Release.

[96] Rostamabadi H., Falsafi S.R., Rostamabadi M.M., Assadpour E., Jafari S.M. (2021). Electrospraying as a novel process for the synthesis of particles/nanoparticles loaded with poorly water-soluble bioactive molecules. Adv. Colloid Interface Sci..

[97] Tanhaei A., Mohammadi M., Hamishehkar H., Hamblin M.R. (2021). Electrospraying as a novel method of particle engineering for drug delivery vehicles. J. Control. Release.

